# NPGREAT: assembly of human subtelomere regions with the use of ultralong nanopore reads and linked-reads

**DOI:** 10.1186/s12859-022-05081-3

**Published:** 2022-12-16

**Authors:** Eleni Adam, Desh Ranjan, Harold Riethman

**Affiliations:** 1 Department of Computer Science, Old Dominion University, Norfolk, VA USA; 2 Medical Diagnostic and Translational Sciences, Old Dominion University, Norfolk, VA USA

**Keywords:** Telomeres, Subtelomeres, Segmental duplications, Tandem repeats, Hybrid assembly, Nanopore, Linked-reads

## Abstract

**Background:**

Human subtelomeric DNA regulates the length and stability of adjacent telomeres that are critical for cellular function, and contains many gene/pseudogene families. Large evolutionarily recent segmental duplications and associated structural variation in human subtelomeres has made complete sequencing and assembly of these regions difficult to impossible for many loci, complicating or precluding a wide range of genetic analyses to investigate their function.

**Results:**

We present a hybrid assembly method, NanoPore Guided REgional Assembly Tool (NPGREAT), which combines Linked-Read data with mapped ultralong nanopore reads spanning subtelomeric segmental duplications to potentially overcome these difficulties. Linked-Read sets of DNA sequences identified by matches with 1-copy subtelomere sequence adjacent to segmental duplications are assembled and extended into the segmental duplication regions using Regional Extension of Assemblies using Linked-Reads (REXTAL). Mapped telomere-containing ultralong nanopore reads are then used to provide contiguity and correct orientation for matching REXTAL sequence contigs as well as identification/correction of any misassemblies. Our method was tested for a subset of representative subtelomeres with ultralong nanopore read coverage in the haploid human cell line CHM13. A 10X Linked-Read dataset from CHM13 was combined with ultralong nanopore reads from the same genome to provide improved subtelomere assemblies. Comparison of Nanopore-only assemblies using SHASTA with our NPGREAT assemblies in the distal-most subtelomere regions showed that NPGREAT produced higher-quality and more complete assemblies than SHASTA alone when these regions had low ultralong nanopore coverage (such as cases where large segmental duplications were immediately adjacent to (TTAGGG) tracts).

**Conclusion:**

In genomic regions with large segmental duplications adjacent to telomeres, NPGREAT offers an alternative economical approach to improving assembly accuracy and coverage using linked-read datasets when more expensive HiFi datasets of 10–20 kb reads are unavailable.

**Supplementary Information:**

The online version contains supplementary material available at 10.1186/s12859-022-05081-3.

## Background

Telomeres are essential for proper replication and stability of chromosomes. They consist of stretches of (TTAGGG) repeat DNA at the ends of chromosomes with associated proteins and at least one lncRNA, TERRA; their dysfunction can contribute to diseases including cancer through multiple mechanisms [1–5]. Subtelomere regions regulate adjacent single-telomere lengths and stabilities of human chromosomes in both telomerase-positive and telomerase-negative contexts [6–9]; thus, accurate maps and DNA sequences for human subtelomere regions, along with detailed knowledge of subtelomere variation and long-range telomere-terminal haplotypes in individuals, are critical for understanding telomere function and its roles in human biology.

Human subtelomere regions remain poorly represented in the widely utilized HG38 human reference sequence and are typically mis-assembled or entirely absent from short-read whole genome assemblies. The principal obstacles to acquiring complete subtelomeric sequences are the abundance of large, highly similar segmental duplication regions and the very high level of structural variation. We have recently developed a short-read assembly strategy based upon identification of Linked-Reads derived from large source DNA molecules extending from 1-copy DNA into segmental duplications, and their assembly to extend high-quality sequence into the segmental duplication regions [10]. Here, we use a Linked-Read dataset from CHM13 to show that subtelomeric assemblies can be improved and extended across a group of representative human subtelomeric segmental duplication regions by combining them with telomere-containing ultralong nanopore reads from the same genome spanning the segmental duplication regions on these subtelomeres. Given the relatively low and rapidly decreasing cost of producing short-read datasets relative to long-read datasets [11–13], NPGREAT may provide an economical route for assembly improvement in contexts where some ultralong nanopore read coverage exists and relatively expensive datasets of deep, high-quality 10–20 kb HiFi genomic reads [11] are unavailable.

## Methods

**Fig. 1:**
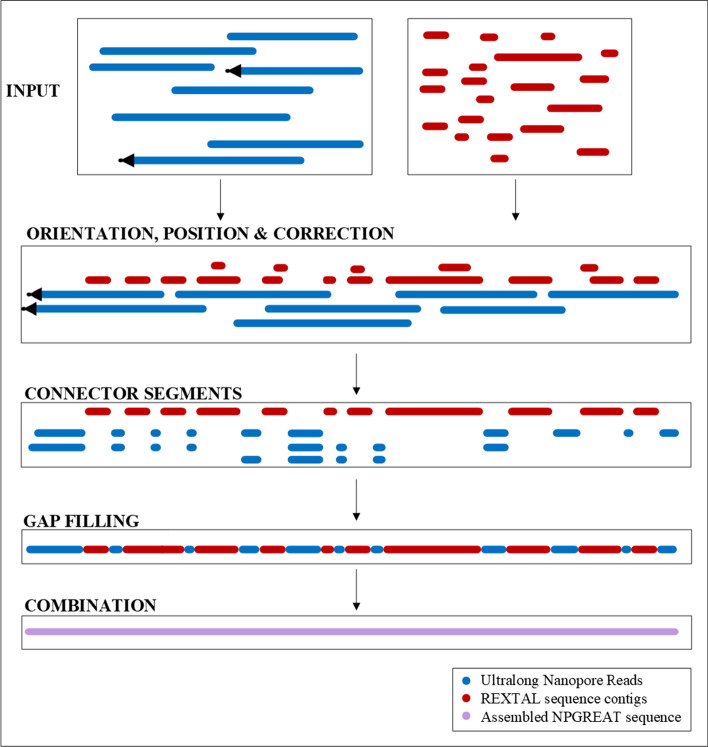
The steps of the NPGREAT method. Telomere-containing ultralong Nanopore reads are shown as blue line segments with black arrows designating terminal ((TTAGGG)n tracts), and are used to anchor the orientation. These along with ultralong Nanopore reads selected from distal 1-copy subtelomere regions are used as scaffolds upon which the Linked-Read assembly (REXTAL) contigs (red line segments) are placed and corrected. The correction of possible misassemblies within the REXTAL contigs is primarily in Tandem Repeat (TR) regions, where the Nanopore reads have a more accurate representation than the short-read Linked-Read assemblies which typically collapse tandem repeats into a short consensus sequence. Properly positioned, oriented, and corrected REXTAL contigs are merged with nanopore connector segments for the NPGREAT output, a single assembled sequence

REXTAL [10] identifies reads corresponding to 1-copy DNA adjacent to segmental duplications, then selects Linked-Reads associated with the identified 1-copy reads from barcode information on the identified reads and assembles all of these reads to extend high-quality assembled sequence from the 1-copy region into the segmental duplication region. A hybrid assembly approach to combine Nanopore reads with REXTAL, called NPGREAT [14] is shown in Fig. 1. It consists of five main operations: Orientation, Position, and Correction, Connector Segments, Gap Filling and Combination. The final output is a single sequence.

### Input

The initial step, prior to the algorithm, corresponds to the selection of the input data, REXTAL contigs and ultralong nanopore reads. To obtain the input REXTAL contigs, we execute the REXTAL procedure [10] and convert its output, which is initially in the form of scaffolds, to unordered DNA sequence contigs. To detect the telomere-containing nanopore reads, we carry out a telomere-tract motif (TTAGGG)n screen on the nanopore read sequence database to select all reads containing this motif at a read end. A read end is defined as a read sequence distal to the last GATC site in a read. The telomere terminal repeat screen requires the presence of 4 consecutive perfect telomere repeats (24 bases) at least twice at the end of a sequence read and was validated initially for human telomere-containing reads from conventional sequence datasets [15]; we used it successfully here for nanopore reads that are basecalled with Guppy version 5.0.7. Although nanopore end (TTAGGG) sequence tracts do contain more errors than conventional and next-gen reads, they are typically also much longer (500–5000 bp, 2221 bp ave length; Additional file 1: Figure S3) than the 50–500 base tracts found in cloned DNA fragments and next-gen reads, and can be readily identified using this screen. A second screen of the same nanopore read database with 1-copy sequences closest to the telomere (Table 1, 10 K 1-copy) results in the selection of subtelomeric nanopore reads containing 1-copy/segmental duplication boundaries; typically, a fraction of these reads extends deep into the subtelomeric segmental duplication region. Telomere motif containing nanopore reads also containing 1-copy boundary reads span the entire subtelomeric segmental duplication region and identify the telomere of origin (mapped telomere read). Mapped telomere and subtelomeric nanopore reads of length greater or equal to 40,000 bases are used as the input nanopore reads of NPGREAT. NPGREAT is designed for analysis of euchromatic subtelomeres, and will likely be unsuitable for heterochromatic subtelomeres (13p, 14p, 15p, 21p, and 22p) where subtelomere-adjacent 1-copy DNA does not exist.

**Table 1:** Coordinates of Subtelomere Regions Analyzed in CHM13 v2.0

Subtelomere region	CHM13 v2.0 reference	Segmental duplication	1-copy region	10 K 1-copy	100 K 1-copy
9p	1–500,000	1–197,765	197,766–500,000	197,766–207,765	197,766–297,765
10p	1–500,000	1–81,456	81,457–500,000	81,457–91,456	81,457–181,456
18p	1–500,000	1–285,485	285,486–500,000	285,486–295,485	285,486–385,485
19q	61,207,364–61,707,364	61,684,066–61,707,364	61,207,364–61,684,065	61,674,066–61,684,065	61,584,066–61,684,065
20p	1–500,000	1–101,050	101,051–500,000	101,051–111,050	101,051–201,050
22q	50,824,926–51,324,926	51,254,087–51,324,926	50,824,926–51,254,086	51,244,087–51,254,086	51,154,087–51,254,086

### Orientation and position

After the input nanopore reads and contigs have been selected, the subtelomeric nanopore reads and the REXTAL contigs are oriented. The orientation of the telomeric nanopore reads is known a priori, given that they always end in the 5′- (TTAGGG)n -3′ telomere tract. Overlapping nanopore reads are then oriented relative to the telomeric nanopore reads and to each other.

REXTAL sequence contigs are aligned, oriented, and positioned relative to the repeat-masked nanopore sequence reads using multiple steps in the REXTAL contig mapping software, including decision steps for potentially conflicting mapping information. Key steps for contig orientation include a minimal alignment threshold of 75% sequence identity for initial contig orientation (with conflicting contig orientation resolved according to the higher Segment Pair score and alignment length with the cognate nanopore read). This is followed by an additional positioning step using only the set of oriented contigs that includes a minimum alignment threshold of 80% sequence identity with the nanopore read. Any resulting reverse alignments (typically short or relatively poorly matching) are removed, as are small contigs positioned completely within larger ones. The positions of the remaining contigs on the cognate nanopore reads are determined by their longest contiguous segments.

### Correction

REXTAL contig alignment with the cognate nanopore read sequence is monitored and alignment discrepancies above a given threshold (typically set at 100 bp) are corrected as described in detail below.

### Connector segments

Alignment of REXTAL contigs with nanopore reads, yield two possibilities for neighboring REXTAL contigs: their overlap or a gap between them. In the connector segments step, the overlapping REXTAL contigs are merged and nanopore read segments that can bridge gaps between neighboring contigs, are identified and extracted.

### Gap filling

For each gap between REXTAL contigs, several nanopore segments may be available to bridge it. In order to fill the gap, we select the segment that has the highest average percent identity with the flanking contigs.

### Combination

In the final step, we combine according to their order the REXTAL contigs, the merged REXTAL contigs and the nanopore selected segments that connect as well as extend them. The result is the assembled sequence.

### Correction steps of NPGREAT

**Fig. 2:**
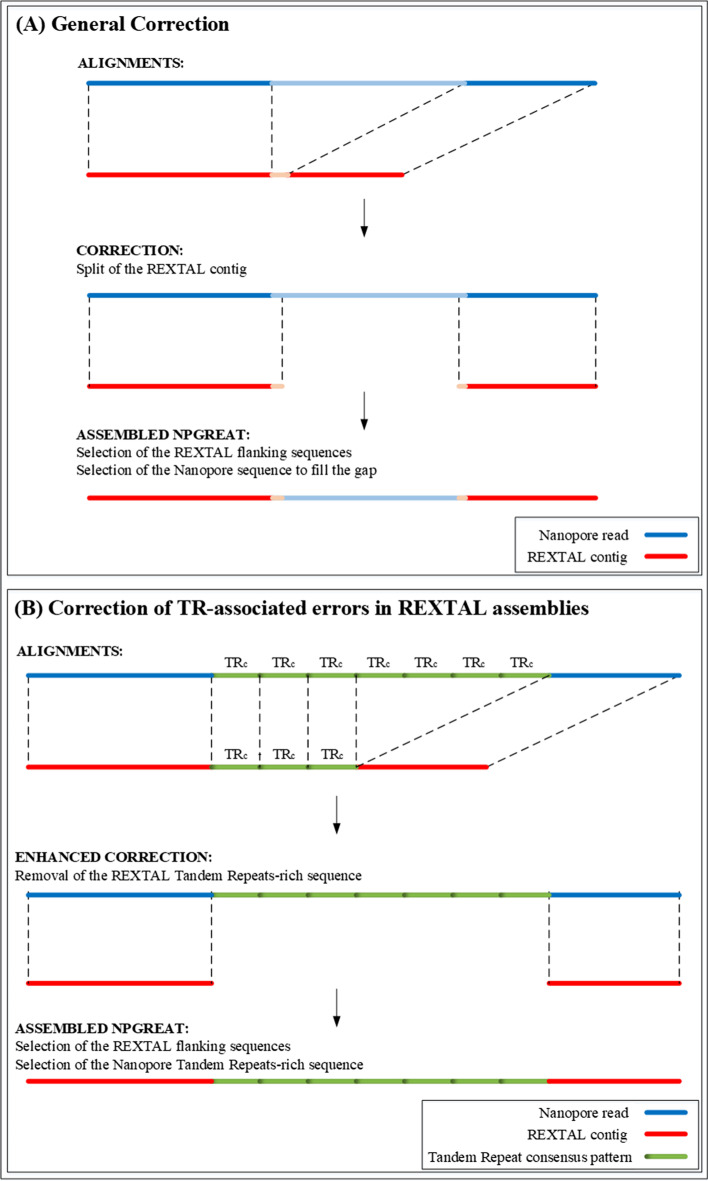
Correction of REXTAL assemblies using Nanopore Reads. **A** The alignments between the nanopore and the REXTAL sequence reveal a segment whose length in the nanopore read (light blue segment) is different than in the REXTAL contig (light red segment). This indicates a potential region where a deletion has occurred in the REXTAL contig. We identify the exact location of the deletion by obtaining the alignments of the deletion boundary regions in question in unmasked mode. Then, we split the REXTAL contig at those coordinates, filling the missing part with the corresponding nanopore sequence. **B** In most cases where length differences between nanopore and REXTAL assemblies are seen, the initial alignments between the nanopore read and the REXTAL contig reveal a tandem repeat region better represented in the nanopore read. In these cases, the tandem-repeat pattern sequence is removed from the REXTAL contig and the contig is split at those junctions. The properly aligned segments from the REXTAL contig flanking the TR region are joined with the nanopore sequence containing the tandem repeat to form the NPGREAT assembly

The correction steps of NPGREAT are able to detect misassemblies within REXTAL contigs and correct them using sequence from nanopore reads. In the correction algorithm, NPGREAT scans the local alignments of a REXTAL contig with the corresponding nanopore read. As seen in Fig. 2A, in the case of a deletion in the REXTAL contig there is a segment in the nanopore read (light blue segment) whose length is much greater than in the REXTAL contig (orange segment); this is identified as a potential need for splitting the REXTAL contig. To localize the deletion within the REXTAL contig, we align the borders of the non-aligned regions of the REXTAL contig (1 kb on each side) in unmasked mode to identify the exact coordinates of the discrepancy. Then, we split the contig at those coordinates and use the nanopore sequence to correct it. For more detail, please see the SPLITS algorithm and figures (Additional file 1: Figs. 4–6).

The threshold of identification can be modulated in the program; a difference in the non-aligned segments above the threshold causes a split in the REXTAL contig and insertion of nanopore-derived sequence to fill the gap. We currently use a 100 bp length threshold, allowing insertion/deletions of short length in REXTAL contigs to be identified. In the case of misassemblies caused by Tandem Repeats (Fig. 2B) unmasked alignment of the two borders (+ 1 kb to the sides), revealed a specific motif, with the beginning and end portions of the nanopore sequence matching well with the beginning and end portions of the REXTAL sequence. However, the middle portion of the REXTAL sequence matched equally well with multiple contiguous parts of the cognate nanopore sequence. These observations indicated the presence of a tandem-repeat region not represented correctly by the REXTAL sequence assembly. The tandem repeat (green color) is identified but its length has been compressed in the REXTAL sequence, whereas the nanopore sequence correctly identifies the length of the tandem-repeat region and positions the repeated pattern in the correct location relative to flanking sequence. Thus, in the tandem-repeat regions, we depend on the nanopore sequence to correctly define the total TR length.

In order to correct the REXTAL contigs that contain misassembled tandem-repeat patterns, we remove the entire repeat pattern region from the contig (middle green region) and simultaneously, split it at the defined borders. The two resulting contigs are aligned with the nanopore read and placed on the sides. In between, the tandem-repeat region will be filled by the nanopore sequence. The correction steps enable an assembly where the representation of misassembled repeats collapsed due to short-read assembly errors is more accurate.

### SHASTA assembly

The SHASTA assembler [16] is used to compute Nanopore-only assemblies. SHASTA assemblies were obtained using the SHASTA assembler version 0.10.0. Based on the features of our dataset, we chose the parameter file “Nanopore-UL-May2022” as recommended in SHASTA documentation. However, in order to incorporate all the desired reads of our dataset, we changed the “–Reads.minReadLength” parameter to 40,000.

An important part of the SHASTA assembly method is creation and use of a “marker graph”. The marker graph that gets created and used during Shasta assembly depends critically on the parameter “–MarkerGraph.minCoverage”. Setting this parameter allows the option of eliminating edges in the graph that have low coverage and might lead to assembly errors. SHASTA’s automatic selection for this parameter for producing high-quality assemblies can be relatively large. However, because of relatively low nanopore read coverage in many subtelomeric regions, especially where there are large segmental duplications, the choice of the automatic selection leads to shorter assembly lengths and consequent gaps in SHASTA assembly coverage adjacent to telomeres.

We therefore use a range of “–MarkerGraph.minCoverage” parameter settings to produce subtelomeric SHASTA-only assemblies for comparison with NPGREAT assemblies, from the automatically selected value to the minimum allowable value. As noted, using the automatically selected value usually results in gaps in the subtelomeric assembly. Using the minimum allowable value provided an assembly, but with suboptimal accuracy due to the low subtelomeric coverage of mappable nanopore reads in the actual CHM13 ultralong read datasets in subtelomeric segmental duplication regions. Details are presented in the Results section of the paper.

### Quast analysis of assemblies

To assess the quality of the REXTAL, NPGREAT, and SHASTA assemblies, we used the QUAST software [17] and the Icarus genome viewer [18]. QUAST is a tool for the pairwise evaluation and comparison of genome assemblies. We used version 5.0, which uses minimap2 as an aligner to align the assemblies to a reference genome, as specified by the user; in our case this was the haploid reference genome CHM13 v2.0 [19].

**Fig. 3:**
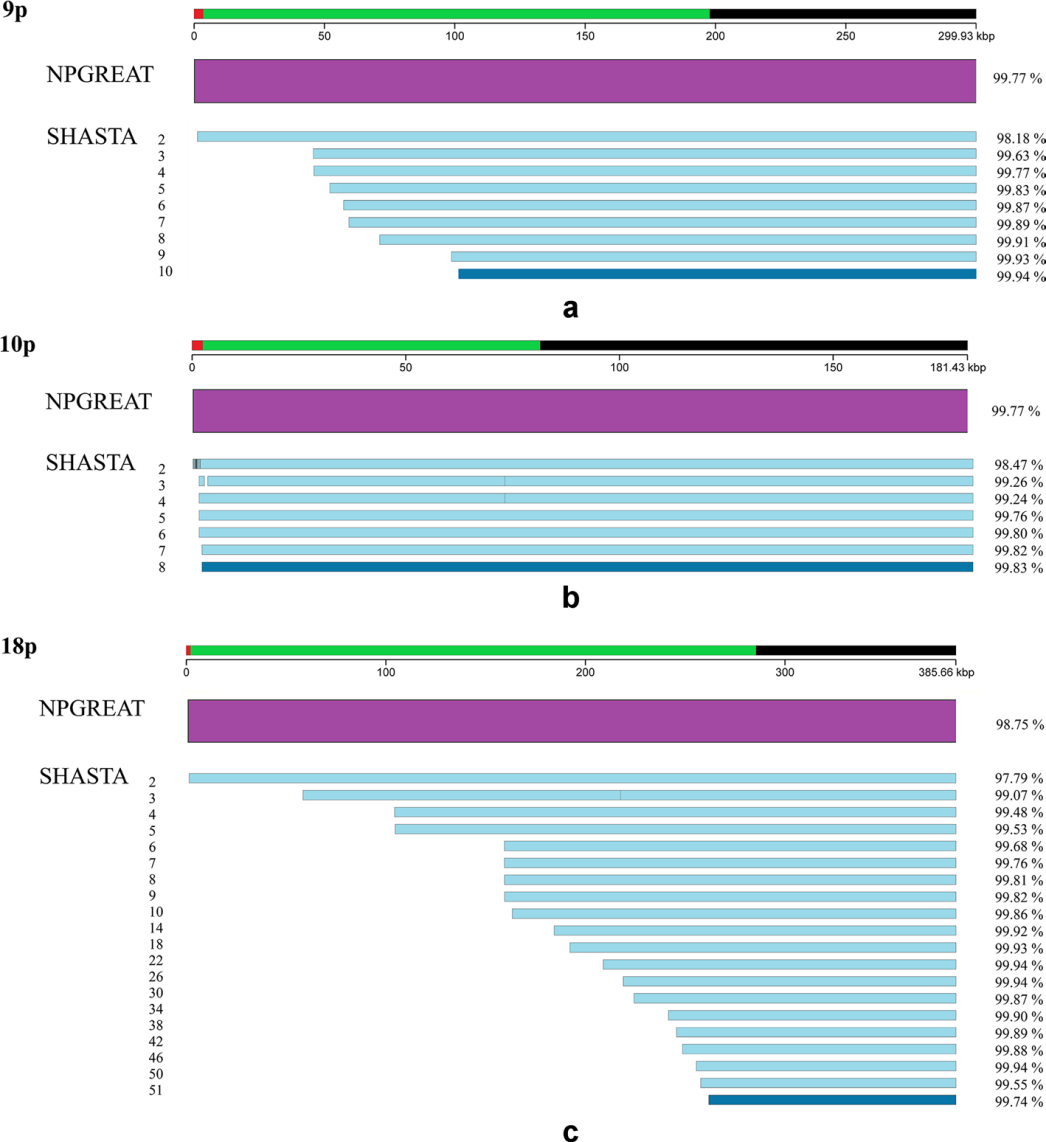

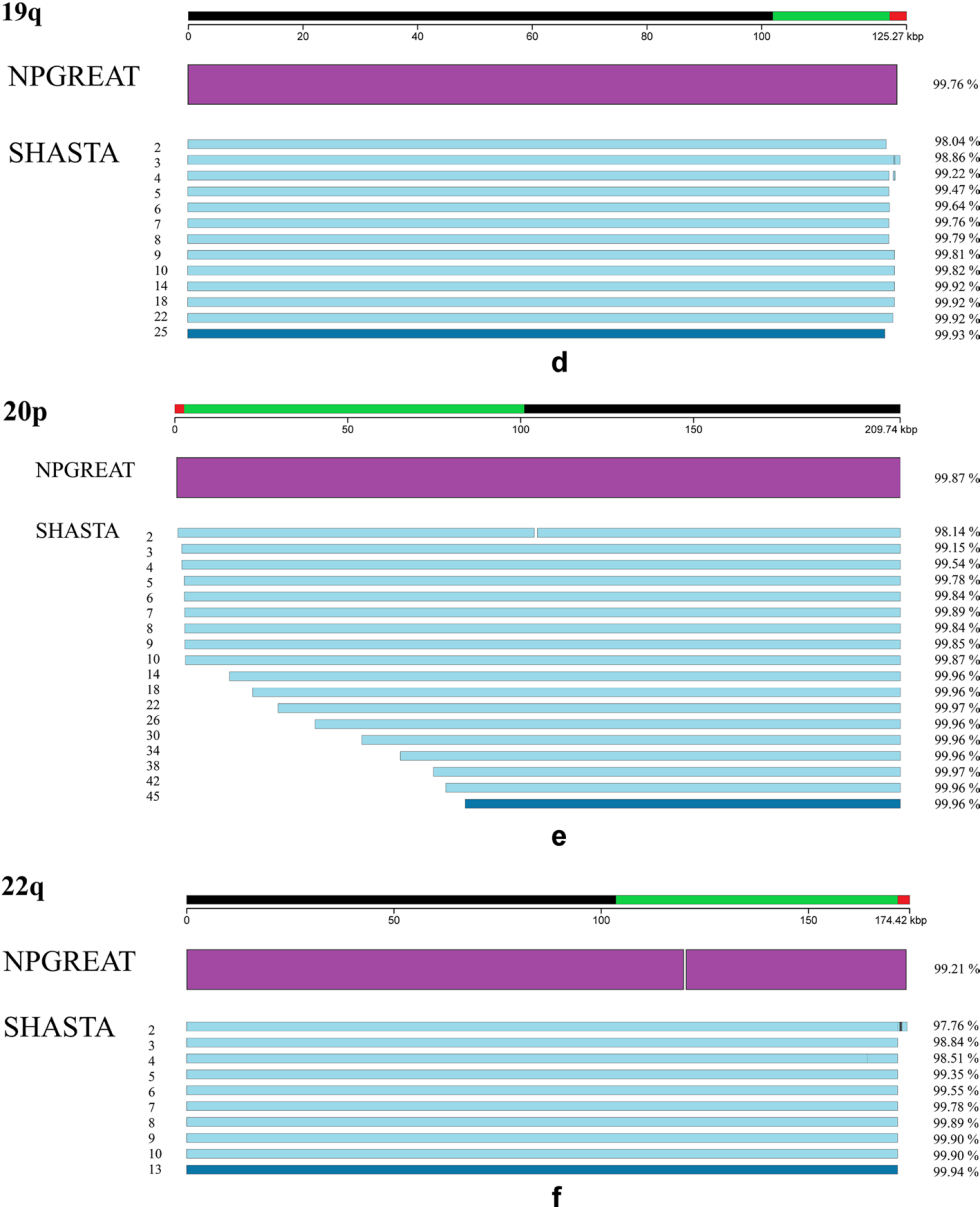
**a**–**f** Comparison of NPGREAT and SHASTA subtelomere assemblies with the CHM13 reference genome using QUAST. To assess the quality of the NPGREAT and SHASTA assemblies for each of the selected subtelomeres, we used the QUAST software [17] and the Icarus genome viewer [18], comparing each assembly with the distal-most regions of the selected telomeres (from the end of the telomere (TTAGGG)n tract through the segmental duplication region and into the start of the 1-copy sequence on the centromeric side of the respective subtelomere) in the CHM13 genome sequence (Fig. 3a–f). For each of these figures, the distal segment of reference sequence is indicated by the line segment at the top of the figure; the telomere (TTAGGG)n tract (red) is represented at the left end of each figure representing the p-arms of chromosomes, and the right end of those figures representing the q-arm, with the segmental duplication regions adjacent to (TTAGGG)n represented in green. The purple rectangle corresponds to the NPGREAT assembly, and the blue line segments below represent the Nanopore-only assemblies using SHASTA set for the indicated coverage parameters. The dark blue line segment for each figure represents the SHASTA assembly using the recommended parameters. Nucleotide sequence similarity of each assembly to the reference sequence segment it is aligned with is shown at the right of the respective assembly.** a** 9p subtelomeric region. There are no misassemblies in either NPGREAT or SHASTA. All assemblies are composed of one contig within this region.** b** 10p subtelomeric region. SHASTA 2, 3, and 4 have misassemblies (designated as vertical gaps in the figure) in the telomere repeat tract area. SHASTA 3 and 4 also have a local misassemblies designated with a vertical line at 73 kb. All assemblies are composed of one contig.** c** 18p subtelomeric region. SHASTA 3 has one local misassembly (designated with a vertical line) at approximately 230 kb. All assemblies are composed of one contig.** d** 19q subtelomeric region. SHASTA 3 and 4 have misassemblies within the telomere repeat tract area; except for these, all assemblies are composed of one contig.** e** 20p subtelomeric region. SHASTA 2 has one local misassembly (designated with a gap at 105 kb). All assemblies are composed of one contig.** f** 22q subtelomeric region. The NPGREAT assembly has a local misassembly designated with a gap at 120 kb corresponding to a LINE/L1 element. The SHASTA 2 has two misassemblies within the (TTAGGG)n tract, and SHASTA 4 has a local misassembly at 164 kb. All assemblies are composed of one contig

We compared each assembly with the distal-most regions of the selected telomeres in the CHM13 genome sequence (Additional file 1: Figure S1 for REXTAL and NPGREAT; Fig. 3a–f for NPGREAT and SHASTA). Using the same haploid reference genome from which the Nanopore and the linked-read libraries were prepared removed ambiguities and apparent misassemblies otherwise caused by normal variation in unrelated genomes and between haplotypes in diploid genomes, providing results much more straightforward to interpret in the context of the assemblies themselves. The total percent identity of each assembly with the CHM13 reference was determined using the percent identities of individual QUAST output alignments. We calculated the weighted percent identity of each assembly as seen in Algorithm 1 (Additional file 1: Figure S2). QUAST can generate multiple contiguous alignments for single assemblies. In these cases, we calculated the weighted average percent identity by using the individual alignment lengths as weights, i.e. multiplying each alignment’s percent identity with its weight, then adding all products and finally, dividing them by the sum of the weights (lengths). In contexts where single alignments are produced for the genome region being analyzed (e.g., many of the QUAST comparisons in Fig. 3a–f), the weighted average percent identity simply corresponds to the percent identity of the single alignment produced. The standard QUAST comparison metrics for these alignments are provided in Additional file 2.

## Results

### Subtelomere assemblies using NPGREAT

We used NPGREAT to assemble Linked-Reads with ultralong nanopore reads of the CHM13 human cell line for the subtelomeric regions of 9p, 10p, 18p, 19q, 20p and 22q, (Table 1), utilizing an ultralong Nanopore reads dataset [20] and a 10× genomics dataset [21]. This set of subtelomeres sample the structural variety of human euchromatic subtelomeres identified by global mapping studies of samples from many human populations [22]. These subtelomeres also represent most types of subtelomeric segmental duplication organizations seen in human populations [22] and are covered by nanopore reads that completely span their subtelomeric segmental duplications in this particular genome (“Telom NP “ in Table 2), extending from (TTAGGG)n repeats into 1-copy DNA for these specific telomeres. Subtel NP are the number of Nanopore reads identified from the 1-copy regions of the indicated subtelomere. As can be seen from Additional file 1: Table S1, mappable nanopore Telom NP read coverage varies greatly across the euchromatic subtelomeres of CHM13, with several subtelomeres with large known segmental duplication regions (1p, 3q, 5q, 6q, 7p, 9p, 9q, 10q, 11p, 16q, 18p, 19p; [15]; [22]) having relatively sparse or no segmental duplication-spanning telomere nanopore read coverage in this particular haploid genome.

**Table 2:** Number of Mapped Nanopore Reads in Subtelomere Regions

Region	Telom NP	SD length	Subtel NP	NPGREAT assembly size (bp)
9p	4	197,765	164	463,841
10p	17	81,456	156	476,942
18p	3	285,485	187	620,639
19q	30	23,299	84	326,420
20p	12	101,050	161	372,980
22q	20	70,840	91	509,481

*Telom NP* (TTAGGG)n -containing nanopore read spanning subtelomeric segmental duplication region, *SD Length* Size of segmental Duplication region adjacent to the telomere. *Subtel NP* nanopore reads selected using distal-most 1-copy probes (100 K 1-copy)

Incorporation of ultralong nanopore reads into the assembly process significantly improved the quality and completeness of the NPGREAT assembly relative to the cognate REXTAL-only assembly, which used only linked-reads. This is readily seen in the QUAST comparisons of NPGREAT assemblies and cognate REXTAL assemblies, respectively, with the CHM13 reference (Additional file 1: Figure S1). The REXTAL contigs designated in red each contain a major misassembly which was detected and corrected by NPGREAT; the resultant NPGREAT assembly no longer contains misassemblies relative to the CHM13 reference. The most common error in the REXTAL-only assembly was an apparent deletion in the REXTAL assembly caused by collapse of reads in Tandem Repeat (TR) arrays in the target sequence (e.g., see the misassembly points co-incident with VNTRs in the CHM13 reference sequence for 18p and 22q in Additional file 1: Figure S1). Misassemblies in REXTAL not associated with tandem repeats were also detected and corrected using NPGREAT (Additional file 1; Fig S1, 9p and 22q QUAST).

### Subtelomere assemblies using SHASTA

In order to compare the NPGREAT assemblies with nanopore-only assemblies from the same CHM13 dataset, the sets of ultralong subtelomeric nanopore reads selected as input for NPGREAT were instead used to assemble these same subtelomeres without any contribution from linked reads by using SHASTA [16]. To test the impact of varying nanopore coverage on the quality and completeness of SHASTA assemblies near telomeres, we assembled the full set of ultralong nanopore reads mapping to each of the six subtelomeres (Fig. 3a–f) using the automatic recommended SHASTA coverage setting as well as a range of more relaxed coverage settings to test how SHASTA would compare with NPGREAT in low-coverage situations expected to typically exist near subtelomeres with large segmental duplications.

To assess the quality of the NPGREAT and SHASTA assemblies, we used the QUAST software [17] and the Icarus genome viewer [18], comparing each assembly with the distal-most regions of the selected telomeres in the CHM13 genome sequence (Fig. 3a–f). QUAST is a tool for the pairwise evaluation and comparison of genome assemblies. We used version 5.0, which uses minimap2 as an aligner to align the assemblies to a reference genome, as specified by the user. Using the same haploid reference genome from which the Nanopore and the linked-read libraries were prepared (CHM13) removes ambiguities and artifactual misassemblies otherwise caused by normal variation in unrelated or even in diploid genomes.

In Fig. 3a–f, we compared each NPGREAT assembly and its cognate nanopore-only assemblies using SHASTA set for a range of coverage parameters with the corresponding distal subtelomere region of the CHM13 human reference sequence [19]. The first line corresponds to the NPGREAT assembly, and the lines below represent the Nanopore-only assemblies using SHASTA set for the indicated coverage parameters. The telomere is at the left end of the figures representing the p-arms of chromosomes, and the telomere end is on the right end of those figures representing the q-arm, as indicated. For clarity, Fig. 3a–f are a simplified view of the QUAST software output.

In each of these subtelomere regions, the NPGREAT assembly has resulted in complete coverage while maintaining a relatively high percent identity with the reference (Fig. 3a–f). The NPGREAT assembly nucleotide sequence percent similarity with CHM13 varied according to subtelomere from 98.75% at 18p to 99.87% at 20p. The input REXTAL contig % similarities were consistently higher than those of NPGREAT, indicating that the expected lower accuracy nanopore sequence read input of nanopore sequence (basecalled using Guppy 5.0.7) contributing to NPGREAT (Fig. 1) decreased the overall % identity of the NPGREAT assembly to some extent, but NPGREAT provided complete coverage of the key telomere-adjacent DNA regions while greatly improving accuracy relative to individual nanopore reads. As expected, NPGREAT assembly regions with nearly complete REXTAL coverage (Additional file 1: Figure S1) had the highest sequence similarity to CHM13.

All of the nanopore reads for each subtelomere (Table 2) were initially included in an assembly using SHASTA under automatic default parameters (See “Methods” section). This assembly is designated with the dark blue rectangle in each of the Fig. 3a–f. The automatic default SHASTA coverage parameters are designed for optimal assembly, and indeed SHASTA performs well in regions with sufficient nanopore read coverage to support it at this level. However, since nanopore read coverage falls significantly as one approaches the chromosome end, especially in subtelomeres with large segmental duplications, SHASTA using this parameter often provides no assembled sequence for these regions. Relaxing the SHASTA coverage parameter increases the amount of assembled sequence produced, but at a cost of lower accuracy relative to the reference sequence; at the most relaxed setting (s), the entire subtelomere is assembled by SHASTA in each case. However, NPGREAT consistently outperforms SHASTA with the more relaxed coverage parameters, providing a higher-quality assembly (Fig. 3a–f).

## Discussion

We show here that NPGREAT can enhance the completeness and quality of subtelomere assemblies relative to Nanopore reads alone. The level of improvement is most pronounced when ultralong nanopore read coverage is relatively low and REXTAL assemblies derived from linked-read libraries provide overlapping coverage of most of the segmentally duplicated nanopore read region. Additional file 1: Table S1 shows the distribution of (TTAGGG)n-containing nanopore reads > 40 kb in size that can be mapped uniquely to euchromatic subtelomeres by virtue of overlap with 1-copy DNA for the CHM13 genome, revealing the large variability in mapped telomeric nanopore read coverage likely due to the size and complexity of segmental duplication regions at specific subtelomeres. Because of the current effort and expense in generating deep ultralong nanopore sequence read libraries, suboptimal nanopore coverage of subtelomeres is likely to remain an issue and resolution and assembly of subtelomeres will require complementary sequencing methods. New methods for generating targeted ultralong nanopore read libraries enriched for telomeric reads at higher coverages would help to alleviate the current difficulties in assembling subtelomeres, but currently complementary sequencing datasets such as those provided by HiFi reads [19] or linked reads such as those required for REXTAL are needed.

NPGREAT is designed as a regional assembly tool, combining REXTAL assemblies using linked-reads (extending from distal 1-copy regions of euchromatic telomeres into subtelomeric segmental duplications) with mapped telomeric ultralong nanopore reads extending into (TTAGGG)n tracts. Because these distal (“bait”) 1-copy regions are present and uniquely identifiable in all genomes, we envision NPGREAT as a supplementary targeted assembly pipeline for improving sequence quality for all euchromatic subtelomeric segmental duplication regions in a given genome, which are highly variable in both size and sequence organization between genomes. In the current proof-of-principle state of development, we relied on existing linked-read datasets and ultralong nanopore read coverage to develop the basic methods and test whether assembly quality could be improved.

While new 10X linked read datasets are no longer being generated, alternative linked-read approaches such as Tell-seq [23] and stLFR [24] technologies produce very similar and robust linked-read datasets that can be utilized by REXTAL. Once established, a regional assembly pipeline for subtelomeres would facilitate assembly of these highly variant and complex segmental duplication regions from any new genome and fill an important gap in our current ability to produce inexpensive and complete subtelomere assemblies for studying telomere function. Along with subtelomere-enriched ultralong nanopore high coverage read libraries, increasing the input molecule sizes used for linked-read library generation would enhance the effectiveness and accuracy of NPGREAT for subtelomere assembly; given the relatively low data generation costs and the recent implementation of new technologies likely to make short-read sequencing much cheaper than it already is [12, 13], coupling linked-read generation with Nanopore technology using approaches such as NPGREAT remains an attractive option for highly accurate sequencing and assembly of subtelomeres, albeit one requiring further development.

A recent study exploring optimal datasets and assembly methods for a diploid human genome pinpointed telomere/subtelomere regions, along with centromeres and high similarity segmental duplications (such as those found at many subtelomeres) as key genomic regions not amenable to automated assembly with current datasets [25]. Even in the haploid CHM13 genome, these regions required extensive manual curation and assembly validation [19]. The recent efforts of Human Pangenome Reference Consortium are paying off with recent analyses [25, 26] pointing the way towards nearly complete haplotype-resolved assemblies. These assemblies are being generated (at great effort and cost) as reference haplotypes, but it is unclear how complex segmental duplication regions of new genomes will be ascertained and compared to the reference haplotypes (assuming all segmental duplication regions are going to be completely resolvable in the pangenome graphs) given the complexity and high variability of these regions.

For example, each individual new genome one wishes to analyze will likely have a unique combination and subtelomeric distribution of complex segmentally duplicated subtelomere alleles for the 82 euchromatic subtelomere alleles in their diploid genome, many of which may be represented in the pangenome but some (perhaps many) may not, including individual-specific alleles. There is strong evidence that these subtelomeric sequences influence telomere length and stability and are thus extremely important biologically, but they have been and still are mostly absent or unassembled in non-Pangenome assemblies. If high-depth coverage in relatively expensive HiFi reads are required for each new genome to even attempt a comparison with the Pangenome reference, this could be cost-prohibitive and impractical, and may be insufficient to provide an accurate comparison/analysis of these genome regions in any event.

New technologies and approaches are clearly necessary to analyze and compare the complex regions such as subtelomeres in uncharacterized genomes, even with the availability of a Pan-genome reference. Far less expensive linked-read libraries could be prepared from new genomes by incorporating inexpensive short-read technologies [12, 13], and perhaps be combined with targeted nanopore ultralong read libraries from telomeric regions of the genome (which would be far cheaper to prepare than full-genome ultralong read libraries). Further development of NPGREAT methods combined with such wet-lab advances could facilitate inexpensive reconstruction of subtelomere segmental duplication genotypes with the assistance of or even independently of reference Pangenome haplotypes in order to place new genome subtelomeric haplotypes in the context of these regions of the existing pangenome assemblies.

The Pangenome haplotypes are just the beginning of the characterization of these complex regions, with new technologies and approaches badly needed to utilize them. These new approaches need to be economical and practical—NPGREAT, with further development and appropriate wet-lab dataset generation/availability, could help achieve this for subtelomeres.

## Conclusions

We present here a hybrid assembly method that combines ultralong nanopore reads with regionally selected Linked-Read assemblies. This NPGREAT method results in extension of high-quality assemblies into otherwise inaccessible segmental duplication regions near telomeres, enhancing our ability to accurately assemble human subtelomere DNA. This information will enable improved analyses of the structure, function, and evolution of these key genomic regions.

## Supplementary Information

**Additional file 1:** Supplementary Figures, Table and Appendix.

**Additional file 2:** QUAST comparison metrics of assemblies in this study.

## Data Availability

The NPGREAT software and the datasets generated/analyzed during the current study are available in the repository: https://github.com/eleniadam/npgreat. The ultralong Nanopore reads (rel8) and the 10 × Linked-Reads for the CHM13 cell line are available by the “Telomere-to-Telomere” (T2T) Consortium at https://github.com/marbl/CHM13.

## Abbreviations

NPGREAT: NanoPore guided REgional assembly tool
TELL-seq: Transposase enzyme linked long-read sequencing
REXTAL: Regional extension of assemblies using linked-reads
tREXTAL: Modified REXTAL to incorporate the TELL-seq linked-reads
SD regions: Segmental duplication regions
TR: Tandem repeat
BLAST: Basic local alignment search tool
BLAT: BLAST-like alignment tool
QUAST: Quality assessment tool for genome assemblies
stLFR: Single-tube long fragment read
HiFi: High fidelity PacBio long reads
