# Supplementary material for: NPGREAT: assembly of human subtelomere regions with the use of ultralong nanopore reads and linked-reads
Source: BMC Bioinformatics. 2022 Dec 16;23:545. doi: 10.1186/s12859-022-05081-3 (PMC9758922; doi:10.1186/s12859-022-05081-3)
Supplement: Supplementary file 1 — Additional file 1: Supplementary Figures, Table and Appendix. [file 12859_2022_5081_MOESM1_ESM.pdf]

Supplementary Figure 1.

QUAST analysis of NPGREAT and REXTAL relative to the CHM13 reference sequence.

To assess the quality of the NPGREAT and REXTAL assemblies, we use the QUAST software and the Icarus genome viewer, comparing each assembly with the distal-most regions of the selected telomeres in the CHM13 genome sequence. Using the same haploid reference genome from which the Nanopore and the linked-read libraries were prepared removes ambiguities otherwise caused by normal variation in unrelated or even in diploid genomes. QUAST is a tool for the pairwise evaluation and comparison of genome assemblies. We used version 5.0, which uses minimap2 as an aligner to align the assemblies to a reference genome, as specified by the user.

The regions are evaluated with the QUAST tool and a minimum acceptable identity of 95% with the reference. In each image, at the bottom view, the assemblies are compared with the entire reference region and at the top, a zoom-in at a specific portion is shown in detail. The telomere end of the p-arm assemblies are on the left, and the telomere ends of the q-arm assemblies are on the right.

Each view consists of three parts: (1) the NPGREAT assembly (top part), (2) The REXTAL assembly (middle part), and (3) the CHM13 v2.0 reference genome region (bottom) to which each of these two assemblies are independently compared using QUAST. The TRs in the CHM13 reference are masked to identify their locations, and appear as gaps in the reference sequence. The colors designate QUAST identified misassemblies relative to the reference sequence. Misassemblies of length longer than 1 kb are designated with red color, while the gray color signifies existence of at least one local misassembly of length less than 1 kb. Green color indicates correctly mapped contigs.

REXTAL misassemblies (red color) have been resolved in NPGREAT. Nanopore sequence enables the correction of the REXTAL contigs, either with the correct identification of the length of Tandem Repeat regions or simply with the split and positioning of the contigs.

9p

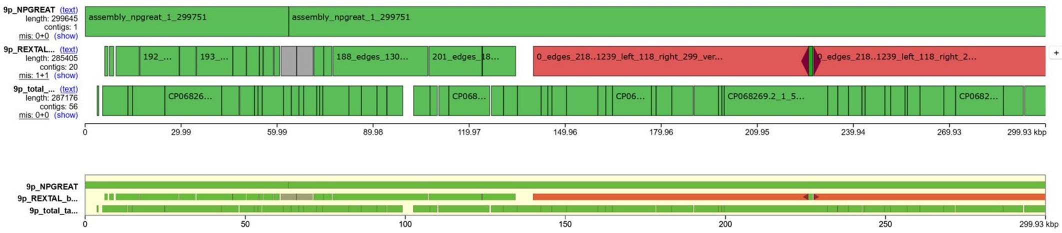

10p

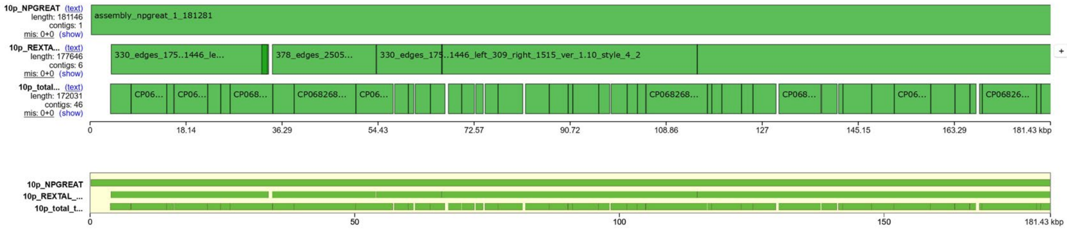

18p

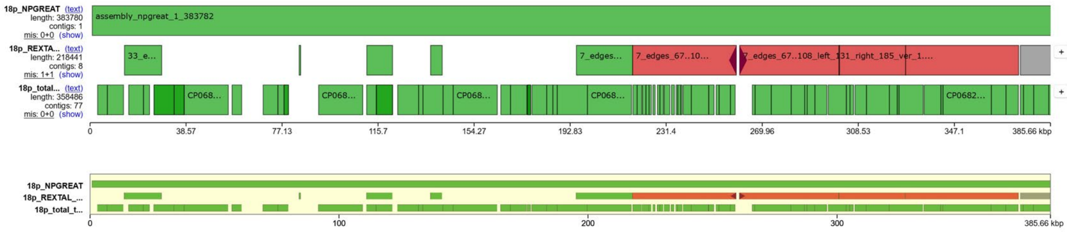

19q

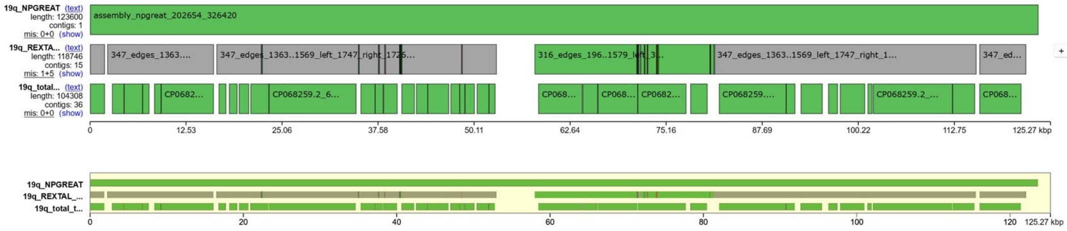

20p

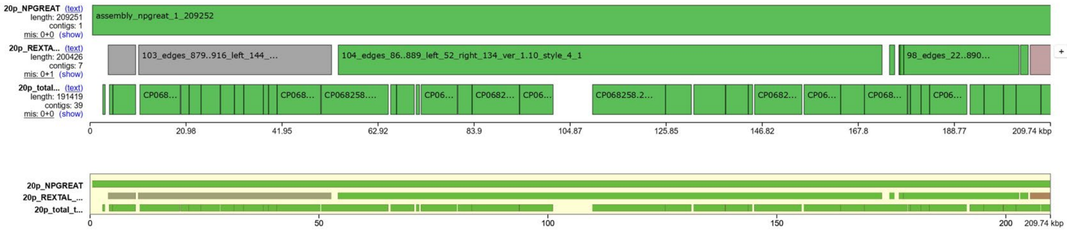

22q

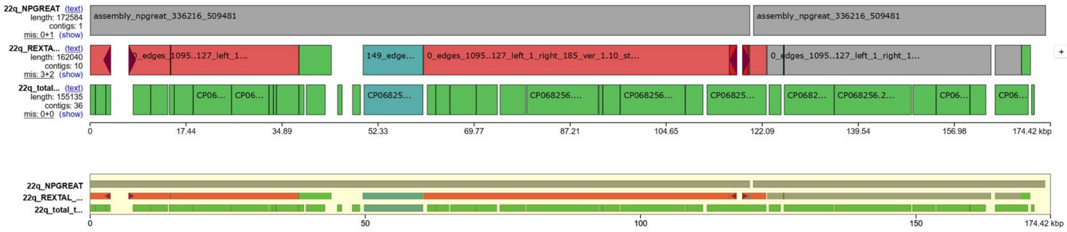

Supplementary Figure 2.

Algorithm 1 – Weighted Average Percent Identity

The total percent identity of each assembly with the CHM13 reference was determined using the percent identities of individual QUAST output alignments. We calculated the weighted percent identity of each assembly as seen in Algorithm 1. QUAST can generate one or more alignments to span an assembled region. We calculated the weighted average percent identity for a given region by using the individual alignment lengths as weights, i.e. multiplying each alignment's percent identity with its weight, then adding all products and finally, dividing them by the sum of the weights (lengths).

Algorithm 1 WEIGHTED_AVERAGE_IDENTITY (Alignments)

```
// Calculation of the weighted average percent identity
// of the Assembly's Quast alignments

flag ← 0

// Search all (sorted) alignments
For each align in Alignments do

        // Check the threshold
        if align.Length < 1000 and flag = 0
                Go to next alignment
        else
                flag ← 1
                // Use the length as weight
                wIdentity ← align.Identity*align.Length
                wsumIdentities ← wsumIdentities + wIdentity

                // Sum of all lengths
                sumLengths ← sumLengths + align.Length

// Calculate the average
wAveIndentity ← wsumIdentities/sumLengths

return wAveIndentity
```

Supplementary Figure 3.

Telomere area length distribution of mapped CHM13 telomeric Nanopore reads.

The number of telomeric nanopore reads (y-axis) containing a telomere repeat tract in the given size ranges (x-axis) is indicated in this histogram.

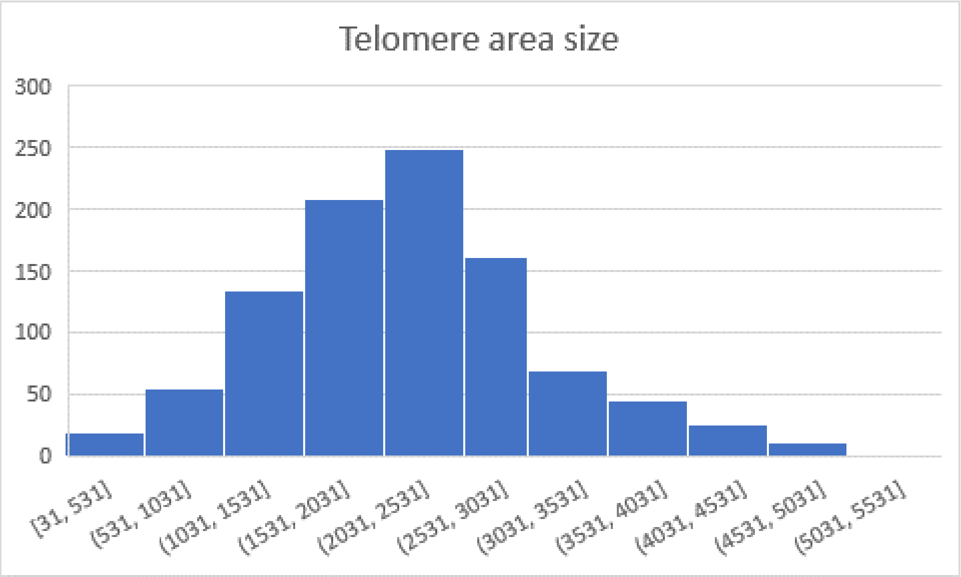

Supplementary Figures 4-6.

Algorithm 2 – Splits

The Algorithm 2 (SPLITS) is part of the correction procedure defining how splits take place. It presents in detail how the Nanopore and Contig areas in question are investigated to detect if a split exists and its exact coordinates. With the analysis of the areas' alignments, a split is detected and the coordinates of the region removal (in case of a TR-associated error) or a cut (in case of a general deletion error) are returned for each area.

Algorithm 2 SPLITS (S)

```
// Input: A set of pairs S = {<xNano, yContig> | xNano: nanopore read segment, yContig: rextal contig segment}.
// Output: The potential split coordinates for the rextal contigs

globalSplits ← {}

// For each pair check possible sequence deletion
for each <xNano, yContig> in S:

      // The set of all alignments obtained by BLAST
      aligns ← blastn (<xNano, yContig>)

      // Alignments sorted based on the nanopore coordinate
      aligns ← sort (aligns)

      // Split coordinates
      split ← EXPLORE (aligns)

      // Add detected split to the list of splits for all areas
      globalSplits ← insert (split)

// For each contig use the procedure SELECT to select the final coordinates of the split
finalSplits ← SELECT (globalSplits)

return (finalSplits)
```

Procedure 1 EXPLORE (aligns)

```
// Input: Alignments of each pair <xNano, yContig>
// Output: Coordinates of the split location, a pair <start, end>. The start and end coordinates of the area to be
// removed.
split ← {}
// Depending on the number of alignments, i.e. size(aligns)
if (size(aligns) = 1):
    // No split
    split ← {}
if (size(aligns) = 2):
    // Removal of Tandem Repeats rich sequence in contig – TR-associated error
    if ((aligns[1].contig[rcoord] > aligns[2].contig[lcoord]) and (aligns[1].nano[rcoord] ≤ aligns[2].nano[lcoord])):
        split ← <aligns[2].contig[lcoord], aligns[1].contig[rcoord]>
    // Removal of Tandem Repeats rich sequence in contig – TR-associated error
    if ((aligns[1].contig[rcoord] > aligns[2].contig[lcoord]) and (aligns[1].nano[rcoord] > aligns[2].nano[lcoord])):
        split ← <aligns[2].contig[lcoord], aligns[1].contig[rcoord]>
    // Deletion in contig detected – General split
    if ((aligns[1].contig[rcoord] ≤ aligns[2].contig[lcoord]) and (aligns[1].nano[rcoord] < aligns[2].nano[lcoord])):
        if ((aligns[2].contig[lcoord] - aligns[1].contig[rcoord]) < (aligns[2].nano[lcoord] –
        aligns[1].nano[rcoord])):
            split ← <aligns[1].contig[rcoord], aligns[1].contig[rcoord]>
if (size(aligns) = 3):
    // Removal of Tandem Repeats rich sequence in contig – TR-associated error
    if ((aligns[1].contig[rcoord] > aligns[2].contig[lcoord]) and (aligns[1].nano[rcoord] ≤ aligns[2].nano[lcoord])
    and (aligns[2].contig[rcoord] > aligns[3].contig[lcoord]) and (aligns[2].nano[rcoord] ≤ aligns[3].nano[lcoord])):
            split ← <aligns[2].contig[lcoord], aligns[2].contig[rcoord]>
if (size(aligns) > 3):
    // Removal of Tandem Repeats rich sequence in contig – TR-associated error
    split ← <min(aligns[2:size(aligns)].contig[lcoord]), max(aligns[1:size(aligns)-1].contig[rcoord])>
return (split)
```

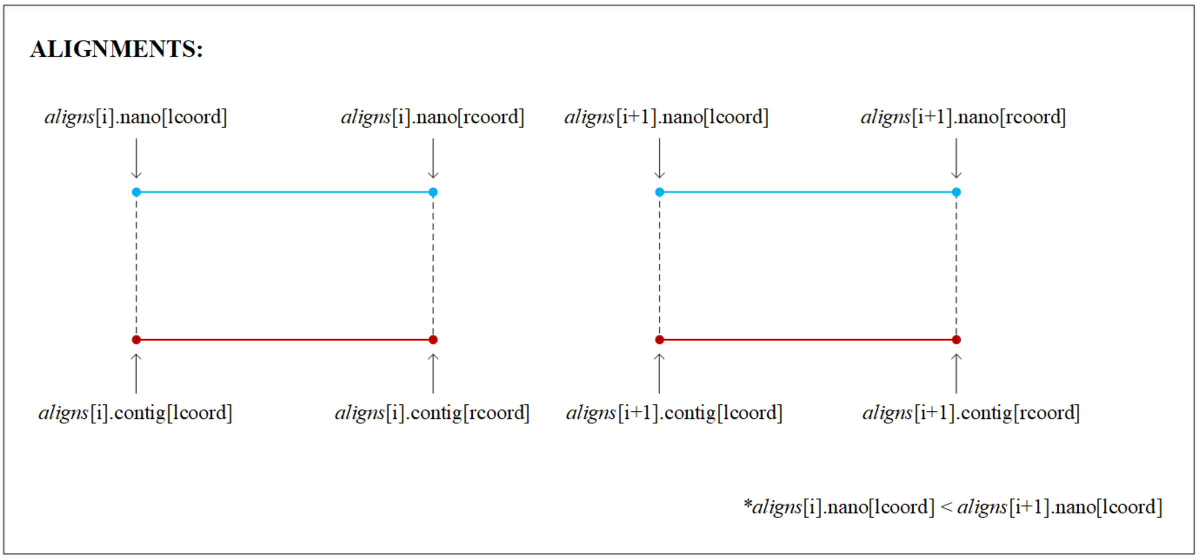

Supplementary Table 1.

Mapped Telomeric Ultra-Long Nanopore reads in CHM13

The number and length of the CHM13 ultra-long Nanopore reads containing the telomere repeat tract $(TTAGGG)_n$ is shown in Supplementary Table 1. For each subtelomeric region ("Subtel"), in column "Telom NP" we indicate the number of the mapped telomeric nanopore reads above 40 kb as identified by the telomeric and 1-copy screens. In the next columns, we identify the number of the reads with lengths greater than 100 kb, 200 kb and 300 kb respectively.

Subtel	Telom NP	>100Kb	>200Kb	>300Kb
1p	0	0	0	0
1q	37	14	3	2
2p	46	16	3	2
2q	10	10	3	0
3p	47	22	8	6
3q	4	4	4	4
4p	23	11	5	3
4q	0	0	0	0
5p	31	12	1	1
5q	3	3	3	0
6p	41	12	7	5
6q	4	4	4	2
7p	5	5	2	1
7q	27	12	2	1
8p	57	26	10	3
8q	37	9	5	1
9p	4	4	4	2
9q	0	0	0	0
10p	17	13	5	1
10q	6	6	6	2
11p	0	0	0	0

11q	35	12	4	1
12p	20	14	2	0
12q	32	14	1	0
13p	Not screened			
13q	48	19	6	2
14p	Not screened			
14q	54	19	5	0
15p	Not screened			
15q	70	70	18	3
16p	28	17	5	1
16q	1	1	1	1
17p	33	13	3	1
17q	37	16	3	1
18p	3	3	3	3
18q	36	13	4	0
19p	3	3	3	1
19q	30	12	2	0
20p	12	12	2	1
20q	11	8	1	0
21p	Not screened			
21q	47	16	6	3
22p	Not screened			
22q	20	17	2	0
Xp	15	11	1	1
Xq	47	16	5	2

Appendix.

The NPGREAT tool requires only one user-specified parameter, the “eval_option”.

- eval_option: Metric for the selection of alignments used for positioning. Set to 0 (default) or 1. eval_option = 0 means that only BLASTn alignments with evalue 0.0 are kept for consideration in the positioning of the reads, otherwise when eval_option = 1 all alignments are kept regardless of the evalue metric.

Using eval_option = 0 is a better option for the majority of subtelomeric areas. However, for some subtelomeric areas, selecting eval_option = 1 is better, given their complexity, e.g. 19q and 22q. For these subtelomeric regions, a lot of the short REXTAL contig alignments have an evalue greater than 0.0 and a big portion of the REXTAL information would be lost if these alignments were eliminated.

For running NPGREAT the following are also required:

- nano_telom_file: The FASTA file containing the telomeric Nanopore reads.

- nano_subtelom_file: The FASTA file containing the subtelomeric Nanopore reads.

- rextal_contigs_file: The FASTA file containing the REXTAL assembly contigs.

- subtel_region_name: The name of the subtelomeric region to assemble in the format

<chromosome ><arm>, e.g. 10p

- repeat_masker_exec: The path to the Repeat Masker executable.

- tandem_repeat_finder_exec: The path to the Tandem Repeat Finder executable.

The NPGREAT tool uses some internal parameters but the user doesn’t have a choice to select the values of these parameters. They were selected with the use of experimentation and empirical optimization, yielding a good dataset for the NPGREAT internal process.

Orientation step:

- The Repeat Masker software with the default parameters: -e rmblast -species human

- The Tandem Repeat Finder software with the default parameters: 2 7 7 80 10 50 500 -f -d -m -h

- The BLASTn software with minimum percent identity: 75

Position step:

- The BLASTn software with minimum percent identity: 80

The minimum percent identity parameters in these steps, were chosen based on experimentation.

Correction step:

- The insertion/deletion identification threshold: 100

- The length of the side sections used in the unmasked alignment mode: 1000

The threshold values in the correction step were chosen based on experimentation.
